# Identifying local-scale wilderness for on-ground conservation actions within a global biodiversity hotspot

**DOI:** 10.1038/srep25898

**Published:** 2016-05-16

**Authors:** Shiwei Lin, Ruidong Wu, Chaolang Hua, Jianzhong Ma, Wenli Wang, Feiling Yang, Junjun Wang

**Affiliations:** 1 Institute of International Rivers and Eco-security, Yunnan University, Kunming, Yunnan 650091, China; 2 Yunnan Key Laboratory of International Rivers and Transboundary Ecosecurity, Yunnan University, Kunming, Yunnan 650091, China; 3 Institute of Yunnan Forestry Inventory and Planning, Kunming, Yunnan 650221, China; 4 Yunnan Academy of Forestry, Kunming, Yunnan 650000, China; 5 School of Ecology and Environmental Science, Yunnan University, Kunming, Yunnan 650091, China

## Abstract

Protecting wilderness areas (WAs) is a crucial proactive approach to sustain biodiversity. However, studies identifying local-scale WAs for on-ground conservation efforts are still very limited. This paper investigated the spatial patterns of wilderness in a global biodiversity hotspot – Three Parallel Rivers Region (TPRR) in southwest China. Wilderness was classified into levels 1 to 10 based on a cluster analysis of five indicators, namely human population density, naturalness, fragmentation, remoteness, and ruggedness. Only patches characterized by wilderness level 1 and ≥1.0 km^2^ were considered WAs. The wilderness levels in the northwest were significantly higher than those in the southeast, and clearly increased with the increase in elevation. The WAs covered approximately 25% of TPRR’s land, 89.3% of which was located in the >3,000 m elevation zones. WAs consisted of 20 vegetation types, among which temperate conifer forest, cold temperate shrub and alpine ecosystems covered 79.4% of WAs’ total area. Most WAs were still not protected yet by existing reserves. Topography and human activities are the primary influencing factors on the spatial patterns of wilderness. We suggest establishing strictly protected reserves for most large WAs, while some sustainable management approaches might be more optimal solutions for many highly fragmented small WAs.

Anthropogenic activities and climate change are causing biodiversity loss at an unprecedented rate^1^. Wilderness areas (WAs), the remaining large intact landscapes with minimal human disturbances, are considered the “last refuges” for a large number of rare and endangered wild animals and plants^2^. WAs also have significant roles in coping with the future threats of human development and climate change, and ensuring the long-term persistence of biodiversity^3^,^4^. However, the remaining WAs are rapidly being lost due to accelerated intensity and scope of human impacts on natural habitats, as a result of the continued increases in commercial production and demands for human livelihoods (e.g., the increasing consumption demands for food, housing, tourism, etc.)^1^,^5^. Therefore, systematic identification of WAs across multiple geographic scales and effectively protecting them have been one of the key strategies for global biodiversity conservation^2^,^3^,^4^,^6^.

Protecting WAs is a crucial proactive approach to ensure the long-term persistence of biodiversity^3^. WAs have been globally accepted as an effective conservation surrogate for establishing biodiversity protected area networks, especially in regions where the spatial distribution data on biodiversity is lacking or sparse^7^. However, many terrestrial WAs are currently still not covered by any type of protected areas^2^,^3^. Usually, such regions are subjected to serious lack of conservation resources in terms of both human and financial capacities^8^,^9^. Moreover, many WAs do not contain high species and ecosystem diversity based on current knowledge^2^, but this does not necessarily mean that biodiversity protection in these WAs is less valuable for a number of reasons. First, the scientific understanding of WAs is still very limited due to the lack of systematic and comprehensive ecological surveys because of their inaccessibility, being located far away from human settlements^10^,^11^. Studies have shown that many endemic species^12^,^13^ and even newly described primates^14^ were found through in-depth investigations in some of the WAs. Second, through maintaining relatively complete ecosystem structures, functions and processes on a large scale^4^, WAs can provide significantly higher value of ecosystem services compared to greatly disturbed regions and their conservation benefit: cost ratio is estimated to be at least 100: 1^15^. Third, WAs often hold the most primitive and unique natural landscapes, which can provide valuable opportunities for many activities, such as scientific research, environmental education, recreation and ecotourism^16^,^17^. Therefore, it is necessary to carry out comprehensive ecological investigations and assessments as soon as possible after identifying the WAs, so as to provide solid scientific basis for the subsequent conservation decision-making along with some potential new scientific discoveries^6^,^16^,^17^.

Wilderness assessments have been widely carried out at broad spatial extents, such as ecoregions^18^, national^17^ and global scales^19^,^20^,^21^,^22^. These studies have played an important role in guiding efficient conservation investments and sustainable land-use planning across large scales. It is generally agreed that natural habitats have been thoroughly affected by human activities, and the few remaining WAs are facing accelerating threats, and hence, call for more actions towards conservation^10^,^22^. However, current large-scale assessments on identifying WAs are too coarse to provide a practical guidance for on-ground conservations^20^,^22^. The results from different studies could vary greatly with each other or even be contradictory in many places at the local scale. For instance, Sanderson *et al*.^22^ found that there were almost no WAs in Three Parallel Rivers Region of China, the core area of one of the global biodiversity hotspots. In contrast, Potapov *et al*.^20^ showed a considerable area of intact forest landscapes remaining in the same region. Such conflicting results from different studies indicate that the large-scale assessments might have left out many important WAs at the local scale that are valuable for implementing practical conservation actions^6^. In fact, the priority-setting studies have shown that priority conservation areas identified at large scales have very limited implications for guiding the local-scale on-ground conservations^23^. Large-scale assessments must be incorporated with the detailed planning of local regions in order to achieve the effective conservation of biodiversity hotspots^23^,^24^,^25^. Caro *et al*.^6^ have clarified the limitations and risks of current large-scale wilderness assessments for biodiversity conservation, and emphasized the importance and emergency of identifying smaller WAs at local extents. In addition, as far as we know, no such study has been reported in China. Therefore, the studies identifying local-scale WAs for guiding on-ground conservation efforts are still very limited.

Here, we aimed to identify the WAs using a systematic planning approach in Three Parallel Rivers Region (TPRR) in southwest China, which is a global biodiversity hotspot as well as a world natural heritage site (Fig. 1). We hypothesized that (1) although humans seem to have altered much of the regions in the world, there were still some remaining WAs at local scales; (2) the current spatial patterns of WAs were most relevant to topographical factors and human disturbances; and (3) most WAs were still not covered by existing reserves. We selected five proxy indicators including human population density, naturalness, fragmentation, remoteness and ruggedness, which were used to characterize the ecological features of WAs and the intensity of human disturbances on natural habitats. We classified TPRR into 10 wilderness levels by implementing a cluster analysis on these five indicators, and the patches characterized by the highest wilderness level 1 and ≥1.0 km^2^ in size were considered WAs. We then investigated the spatial distribution patterns of different wilderness levels and WAs, and finally assessed the conservation status of WAs within existing nature reserves (NRs).

## Results

### Spatial patterns of wilderness levels

In general, the northwest regions showed significantly higher levels of wilderness compared to the southeast. The areas with high wilderness category (level 1–3) in the northwest were large in size and well connected, while those in the southeast were severely fragmented in small patches (Fig. 2). The wilderness level in valleys was generally much lower than that of the adjacent high mountain areas. In addition, the wilderness level in Nu-Salween valley was significantly higher than that in Lancang-Mekong and Yangtze valleys (Fig. 2). Furthermore, the high wilderness category accounted for 43.6% of TPRR’s total area, 43.0% and 31.6% of which were distributed in Diqing and Nujiang, respectively. On the other hand, 32.0% of TPRR’s land was categorized as intermediate wilderness (level 4–7), 88.1% of which was distributed in three prefectures including Diqing (37.1%), Dali (27.5%) and Lijiang (23.5%). Meanwhile, the low wilderness category (level 8–10) accounted for 24.4% of TPRR’s total area with 68.0% found in two prefectures including Dali (39.8%) and Lijiang (28.2%; Fig. 2). The area proportions of high wilderness category covering the total area per prefecture decreased in the order of Nujiang, Diqing, Lijiang and Dali, while the proportions of intermediate and low categories ascended in the same order (Fig. 3a).

Wilderness levels significantly increased with the increasing of elevation (Spearman’s Rank Correlation *r* = −0.569, *n* = 10,000, *P* < 0.001). Accordingly, the area ratios of high wilderness category in different elevation zones continuously increased with elevation, while those of the low category showed an opposite trend (Fig. 3b). In particular, the area ratios of high wilderness category in elevation zones of above 3,000 m were all greater than 51%, but no low wilderness category was distributed in regions higher than 4,500 m. In regions between 2,500 and 3,500 m, the proportion of intermediate category was relatively higher and reached around 35%. For elevation zones lower than 3,000 m, the area ratios of low category were all greater than 27%, and reached 58% in the zone of lower than 1,500 m (Fig 3b).

The mean coverage of high wilderness category in the 23 vegetation types was 49.6% ± 5.9% (range = 0.2–94.9%). In particular, the area proportions were all greater than 40% in the 13 vegetation types, including ice/snow (ICE; 94.9%), rock (ROC; 94.3%), bamboo forest(BF; 86.9%), alpine scree (AS; 86.2%), monsoon evergreen broad-leaf forest(MEBF; 79.8%), mid-montane humid evergreen broad-leaf forest (MHEBF; 69.5%), cold temperate shrub (CTS; 68.4%), mixed conifer broad-leaf forest (MCBF; 64.8%), alpine meadows(AM; 61.2%), temperate conifer forest (TCF; 56.1%), deciduous broad-leaf forest (DBF; 55.4%), semi-humid evergreen broad-leaf forest (SHEBF; 48.7%) and sub-alpine meadow (SAM; 42.0%). In vegetation types like cold temperate montane hard-leaf evergreen oak forest (CTMHEOF), grassland (GRL), warm conifer forest (WCF), dry/hot valley hard-leaf evergreen oak forest (DHVHEOF) and lakes (LAK), the area ratios of intermediate category were higher and all greater than 35%. At the same time, in warm temperate sparse tree shrub (WTSTS), dry/hot shrub (DHS), agriculture (AGR), artificial building (ABD) and rivers (RIV), the low wilderness category dominated and accounted for over 40% of the total area in each type (Fig. 3c).

### Spatial patterns of WAs

The WAs occupied a total land area of 16,468.9 km^2^, accounting for 24.6% of TPRR’s total area. It consisted of 301 patches with various sizes ranging from 1.0 to 1,837.6 km^2^. The number of patches that were smaller than 50 km^2^ accounted for 83.3% of the total number, but they only made up 9.4% of the total area of WAs. Whereas, even if the patches that were greater than 300 km^2^ accounted for only less than 4% of the total patch number, their total area on the other hand composed 60.2% of the total WAs (Fig. 4a). WAs were mainly distributed in Diqing and Nujiang which held 52.4% and 35.6% of the total area of WAs, respectively, followed by Lijiang (9.2%) and Dali (2.8%). In terms of the area ratio of WAs covering the total land area per prefecture, the highest was observed in Nujiang (40.2%) followed by Diqing (37.2%), Lijiang (11.2%) and Dali at 3.0% (Fig. 4b). WAs covered 47.5% of the regions over 3,000 m, where held 89.3% of the total area of WAs, while only 0.46% of WAs was located in areas below 2,000 m (Fig 4c). WAs covered 20 vegetation types in total, with TCF, CTS and alpine ecosystems (i.e., SAM, AM and AS) being the major types, which accounted for 41.9%, 23.2% and 14.3% of the total WAs area, respectively. Some other vegetation types including DBF, BF, SHEBF, ROC, MEBF and GRL, each of them covered less than 1% of the total WAs (Fig. 4d). There were 13 vegetation types, each of which had more than 20% of its total area covered by WAs. Although each of MEBF, DBF and BF only accounted for a very small portion of WAs, WAs covered more than 23% of the total area of each of these three vegetation types (Fig. 4d).

The spatial distribution of our WAs was generally consistent with that of the intact forest landscapes and plant diversity priority conservation areas (Fig. 5). The overlapping areas accounted for 75.6% and 60.3% of the total areas of intact forest landscapes and plant diversity priority conservation areas, respectively. In addition, we also identified many new WAs including (1) the large areas of TCF and MHEBF ecosystems located in Meili Snow Mountains, south Biluo Snow Mountains, and south Gaoligong Mountains, (2) many patches in TCF, CTS and alpine ecosystems distributed from Shangri-La to northeast Yulong and north Ninglang, and (3) a large number of severely fragmented and small-sized patches in TCF, CTS, MCBF and alpine ecosystems extensively distributed from TPRR’s central to southeastern areas (Fig. 5). Moreover, some of the WAs that were continuously distributed in large areas in previous studies exhibited as more fragmented and small patches in this study, such as Jiawu Snow Mountains, Qianhu Mountains, Cangshan Mountains, among others (Fig. 5).

### Conservation status of WAs

TPRR included four national NRs and eight provincial NRs (Fig. 5) with a total area of ~8,517 km^2^, covering 12.7% of TPRR’s land. Existing NRs covered 4,824.3 km^2^ of WAs, which accounted for 29.3% of the total WAs area and 56.6% of the total NRs area, respectively. Of the 301 WAs patches, 35 had over 17% of their land protected within NRs and other six received protections ranging from 1.2% to 12.2%. However, there were still 260 patches that were not covered by any NR. The protection rates of WAs patches with areas larger than 10.0 km^2^ were much higher than those smaller ones. In particular, the overall protection rate of three WAs patches with areas larger than 1,000.0 km^2^ was 76.1% (Fig 6a). WAs located in Nujiang had the highest protection rate (46.2%), followed by Diqing (21.7%), Dali (16.0%), and Lijiang (11.4%; Fig. 6b). The protection rates of WAs located in different elevation zones were all over 17%, and had a decreasing trend with increasing elevation (Fig. 6c). Among the 20 vegetation types located within WAs, 11 had over 17% of their total area protected, and four of which exceeded 50%. Additionally, MEBF had the highest protection rate reaching 82.9% (Fig. 6d).

## Discussion

### Spatial patterns of wilderness in TPRR

In general, the northwest areas characterized by high mountains and deep gorges had significantly higher wilderness levels in comparison with the relatively flat southeast regions (Fig. 2). This spatial pattern of wilderness levels was generally consistent with the results of previous global- or local-scale wilderness assessment or conservation planning studies^20^,^22^,^24^. The northwest with TCF as a dominant vegetation type is the major distribution area of natural forests in TPRR. Whereas, in the southeast, the current vegetation types dominated by WCF are mostly secondary forests formed through afforestation or natural succession after the destruction of primary vegetations^26^,^27^. Thus, the naturalness in the northwest is much higher than that in the southeast. The extremely steep and rugged terrain in the northwest also makes it difficult for many human exploitation activities such as land development and natural resources extraction^28^. Moreover, the northwest has a relatively low human disturbance level characterized by low human population density and low development intensity of roads, cities, industries and agricultures, with the majority of such developments located along both sides of the great rivers. Thus, human activities mainly put clear impacts on the ecosystems near valleys or adjacent to main roads^29^.

In contrast, the southeast has been historically the center of regional economic development for a long time due to its more favorable environmental conditions for human existence such as relatively flat topography, warmer climate and fertile soils^27^,^28^. Therefore, the intensity of human disturbances to natural ecosystems is much higher in the southeast compared to the northwest, which results in more severe habitat fragmentation in the former. These are the key driving forces that have formed the overall spatial pattern of TPRR’s wilderness. Accordingly, in terms of the administrative units, wilderness levels decreased in the order of Nujiang, Diqing, Lijiang and Dali (Figs 2 and 3a).

Population density is a good indicator of the intensity of human disturbances on natural ecosystems^2^,^10^,^22^. The spatial distribution of human population density in TPRR is negatively and linearly correlated with elevation^30^. Accordingly, the intensity of land development, as the major mechanism that humans affect natural environment^1^, exhibits a clear vertical pattern on the elevation gradient^31^. For example, it has been previously shown that agricultural activities are one the major driving forces causing the fragmentation and loss of natural habitats in TPRR, and the upper elevation limit for agriculture is approximately 3,000 m^31^. Natural conditions are getting worse for human living as the elevation rises (e.g., increased difficulty with getting water)^30^, and particularly in areas above 3,000 m, human disturbances are obviously reduced and land-use patterns usually remain stable for a long time period^31^. Thus, wilderness levels clearly increased with elevation rises (Fig. 3b).

The ecological integrity of each vegetation type is an integrated reflection of the effects of historical human activities^5^,^10^. This is exactly the case in TPRR. For example, WCF is the major forest type developed through the Farm-to-Forest program; WTSTS is mainly formed after severe human disturbances on primary forests such as deforestation, wildfire and grazing; and large areas of GRL are the major places for grazing^26^. Some vegetation types located at valleys and low elevation areas in the southeast (e.g., CTMHEOF, DHVHEOF, and DHS) have low values for biodiversity conservation due to high human disturbances^32^. This study also showed that these vegetation types were dominated by intermediate to low wilderness levels (Fig. 3c). Whereas, evergreen broad-leaf forests, alpine ecosystems, TCF, MCBF and CTS hold a large number of rare, endangered and endemic plant species, and maintain large areas of high-quality natural habitats due to their remote location in higher elevations with less human disturbances^24^,^32^. Moreover, TCF is one of the major vegetation types of the remaining primary forest ecosystems in TPRR, and it provides an irreplaceable habitat for many important plants and animals (e.g., Yunnan Snub-nosed Monkey, many Orchids and Rhododendrons)^33^. TCF and DBF are also recognized as the representative vegetation types of natural forests in TPRR^34^. Although only having a very small distribution range, MEBF has the highest conservation value among all vegetation types because it harbors extremely high number of rare, endangered and endemic species, and TPRR is the northern margin of its geographical distribution^24^,^32^,^34^.

Due to its extreme elevation and climatic conditions, alpine ecosystems host large number of rare and endemic plant species, and also provide important habitats for many alpine animals. In addition, they are of great significance to the local (especially Tibetan) traditional cultures and livelihoods by providing foods, medicinal plants and pasture lands. However, alpine ecosystems are highly vulnerable to the impacts of human activities and climate change^13^. Therefore, these ecosystems are widely recognized as key conservation targets in TPRR^34^. It is worth noting that SHEBF has been severely exploited because the oak wood in this vegetation is the main fuelwood used by local residents, and an effective conservation and management are still lacking^26^,^27^. Most river and lake ecosystems have low wilderness levels because they are often adjacent to urban areas or industry centers, and are also heavily affected by tourism and many construction projects for irrigation and hydropower^35^. For instance, it may be primarily due to the non-point pollution from nearby cities and villages and the impacts of tourism development, the ecological functions of Erhai Lake and Lugu Lake have been significantly degraded^36^.

Approximately 88% of the total area of WAs was located in Nujiang and Diqing, where most patches were large and well connected, while Lijiang and Dali only included small portions of WAs distributed in highly fragmented and isolated small patches (Figs 4b and 5). The large WAs patches were mainly located in places with high elevations (>3,000 m), rugged terrains and less human disturbances. These places included but not limited to Gaoligong Mountains, Meili Snow Mountains, Biluo Snow Mountains, Chali Snow Mountains, Baima Snow Mountains, north Shangri-La, Napa Lake, Bita-Shudu Lake area, Qianhu Mountains, Haba Snow Mountains, Yulong Snow Mountains, Laojun Mountains, Mianmian Mountains, and Cangshan Mountains (Fig. 5). They are suitable for establishing reserves with strict protection as the primary management objective (e.g., IUCN Protected Area Management Categories Ia, Ib and II)^37^, so as to protect a large area of intact natural ecosystems in TPRR and to sustain many critical ecosystem services. We observed that TCF, CTS and alpine ecosystems were the dominant vegetation types in WAs, approximately making up 80% of the total WAs (Fig. 4d). Although some vegetation types only occupied very small ratios of the total area of WAs, the proportion of each of them being classified as WAs was high due to their small total areas. This indicates that these vegetation types have relatively high wilderness levels and conservation values, such as MEBF, DBF, MCBF, BF, MHEBF, etc. (Fig. 4d).

Previous studies estimated that the coverage of various priority conservation areas in the biodiversity hotspot of Mountains of Southwest China ranged from 8% to 93.8%^5^,^20^,^21^,^22^,^29^, which indicates a great uncertainty. In particular, when compared with the results of Sanderson *et al*.^22^ and Potapov *et al*.^20^, this study found a larger area of WAs. This discrepancy with that of Sanderson *et al*.^22^ should be associated with the difference in study scales. Their study was conducted at a global scale, and thus the proxy indicators and datasets they used might not necessarily be applicable to local settings^5^,^22^. For example, Sanderson *et al*.^22^ set the influence distance of road as 15 km when calculating for accessibility, which is obviously unreasonable for regions characterized by high mountains and deep gorges like TPRR. Meanwhile, Liu *et al*.^29^ only used 1.0 km in their study for assessing the ecological impacts of road networks in TPRR. In addition, we also revealed a large number of WA patches of <50 km^2^, while previous studies only identified larger patches of 500 km^2^ ~4,000 km^2^ and mostly focused on forests^19^,^20^. For instance, although shrubs, meadows and grasslands are some of the key ecosystems in TPRR, which also have high conservation values, Potapov *et al*.^20^ did not conduct detailed study on these types.

The spatial patterns of our WAs were more similar to the plant diversity priority conservation areas proposed by Ma *et al*.^24^. However, some large patches of the plant diversity priority conservation areas were found to be highly fragmented in this study, such as Jiawu Snow Mountains, Qianhu Mountains and Cangshan Mountains (Fig. 5). This might be because we have conducted spatial analyses using more detailed datasets on road networks and land uses, which helped reveal the internal structure of each patch with more scrutiny. Studies have shown that even a small road is able to break the ecological integrity of natural habitats adjacent to it^38^. Moreover, in recent years, the tourism development and other economic activities in both Qianhu and Cangshan Mountains have resulted to apparent destruction of their ecological environment and increased fragmentation^27^,^28^,^39^.

### Conservation status of WAs

NRs coverage in TPRR (12.7%) is much higher than the mean coverage of 7.4% in Yunnan^40^ and also significantly higher than that in all the other provinces except Tibet, Qinghai, Sichuan, Gansu, Heilongjiang and Xinjiang^41^. This emphasizes the important role of TPRR in conserving biodiversity for Yunnan and even the whole country. However, there is still a large gap between TPRR’s protection rate and CBD’s 2020 Target of “protecting at least 17% of terrestrial and inland water areas”^42^. By systematically identifying WAs, analyzing their spatial patterns and investigating the influencing factors, this study should be able to provide an important basis for future expansion of TPRR’s NR system. The fact that WAs accounted for 56.6% of the total NR area signifies that wilderness was often implicitly used as an important reference in establishing NRs though WAs have not been explicitly and systematically identified before. Considering the high importance of WAs in biodiversity conservation, the current NR coverage (29.3%) is far from enough, and hence, WAs should be given high priority for establishing new NRs.

As the strictest type of protection level of landscapes in China, NRs tend to be established in regions with high elevations, high ecological integrity and large patches of natural ecosystems^43^,^44^. Large WA patches can better maintain the integrity of the structures, functions and processes of natural ecosystems so as to provide more effective habitats for the long-term persistence of wild species^3^. In addition, they usually have low conservation costs and are easy to manage due to low human disturbance intensity^2^,^8^,^9^. Our results also showed that WA patches in larger sizes exhibited significantly higher NR coverage compared to those smaller patches (Fig. 6a), and the highest NR coverage for WAs was allocated in the large patches of Nujiang followed by Diqing (Fig. 6b). Currently, however, only a few large WAs patches have achieved higher NRs coverage. These NRs include Gaoligong Mountains National NR, Baima Snow Mountains National NR, Yulong Snow Mountains Provincial NR and Haba Snow Mountains Provincial NR (Fig. 5). There are still many large WAs as well as the vast majority of fragmented and isolated patches that are not covered yet by any NR (Fig. 5).

Previous studies revealed that despite their size, small fragmented habitats still play a crucial role in sustaining biodiversity, and can provide significant habitat refuges and dispersal corridors for many species like endangered plants, birds, amphibians, and reptiles^45^,^46^. We previously also showed that the fragmented WAs between Nujiang, Ljiang and Dali provide habitats for five groups of Yunnan Snub-nosed Monkeys (*Rhinopithecus bieti*) – a globally critically endangered primate species^33^. These small WAs should also play a crucial role in providing critical ecosystem services to local communities, such as water supply and soil retention^47^,^48^. However, due to the high intensity of surrounding human disturbances, conservation of these small fragmented WAs might not only be limited to strict NRs.

Detailed analyses on the conservation status of vegetation types that are located within WAs can provide more substantial insights useful as guides for future expansion of protected areas. For example, we found that the distribution range of four vegetation types (i.e., MEBF, BF, DBF and MHEBF) within WAs were well captured by NRs with over 50% coverage, while NR coverage was relatively lower for AS, SHEBF, AM and SAM (Fig. 6d). Considering the high conservation significances of WAs as well as their natural vegetation, we suggest that future expansion of NRs should focus on the WAs that contain some natural vegetation types with less than 30% NR coverage.

### Our analyses

Given the complex topographical conditions of extremely high mountains and deep gorges in TPRR, the five indicators we used could well characterize the spatial patterns and the main influencing factors of the wilderness. For example, remoteness calculation has considered multiple factors on distance, roads, terrain and land cover types, which allowed us to achieve a more authentic measure of accessibility in such complex topographies. Compared with previous researches, this study revealed the spatial patterns of WAs in greater detail by using datasets with higher accuracy (e.g., vegetation types, land-use types, roads, and DEM), so as to provide more reliable support for practical conservations. Our analyses were explicitly driven by the spatial data layers on five indicators, and then applied a cluster analysis to derive the wilderness levels and WAs, which helped minimize the subjectivity of relying on expert knowledge. This ensured that our analyses were relatively transparent, replicable, objective and rigorous. In addition, our approach used publicly available datasets, and was transferable and easy to implement, and should also have significant applications to other global biodiversity hotspots in other parts of the world for enhancing their own on-ground conservation efforts.

However, we acknowledge that the availability and accuracy of spatial datasets play primary constraints in WAs identification. The results should be constantly updated as more comprehensive data become available in order to provide a solid support for conservation decisions. This study did not include the human impacts of grazing, non-timber forest products collections (e.g., food, mushroom and medicinal plants) and mining due to lack of spatial datasets on these activities. Impacts of such activities on many natural ecosystems are growing because of the increases in the local communities’ livelihood and market demands. Thus, comprehensive mapping of the spatial distributions of human activities and systematic investigation of their ecological impacts are future research priorities for enhancing methods in identifying WAs. Moreover, climate change has been demonstrated to have apparent impacts on some ecosystems in TPRR, particularly revealing that the alpine environment is the most susceptible^13^,^49^.

### Conservation implications

It is well acknowledged that protecting WAs is a fundamental conservation priority and crucial for achieving the most cost-effective conservation^6^. TPRR is one of China’s last few areas that still retain a relatively large intact landscape^39^. However, this region is experiencing increasing pressures from various economic activities, such as agricultural and urban expansion, grazing, road construction, tourism, mining, and hydropower development^27^,^35^. The WAs identified in this study could provide spatially explicit guides for preventing such economic activities from further damaging the high biodiversity significant areas. Most WAs should be prioritized for establishing strict reserves (e.g., NRs), particularly those containing large patches, located in high elevations and surrounded by low human population density. However, our results indicated that there are still existing large gaps in the conservation of these large WAs (Fig. 5). For instance, no NR is established along the entire mountain range that extends from Meili to Biluo Snow Mountains. For many WAs, very few surveys have been conducted and our scientific understanding is thus quite limited. Preserving these areas will ensure the sustained future opportunities for some new vital findings, such as new plant and animal species. Nevertheless, it is also unrealistic to strictly protect all WAs. We suggest that many fragmented and isolated small WAs be subjected to a variety of sustainable management approaches with the primary goal of achieving long-term conservation and management, such as the IUCN Protected Area Management Categories V and VI^37^, and the various ecosystem service policies implemented in China^50^.

## Conclusions

Protection of WAs is greatly significant to maintain the long-term persistence of biodiversity in the face of accelerating anthropogenic threats and climate change. This study assesses the wilderness levels and identifies a fine-scale portfolio of WAs for guiding the on-ground conservation actions in a global biodiversity hotspot. The remaining WAs cover about one quarter of TPRR’s total land. Northwest areas exhibit much higher wilderness levels than the southeast. WAs located in the northwest, high elevations and remote areas are relatively large in size and well connected, while most WAs in the southeast and low elevation regions are very small and highly fragmented. Topography and human activities are the primary influencing factors on the spatial patterns of wilderness. Most WAs are still not covered by existing NRs. We suggest different approaches in conserving WAs depending on their locations and characteristics. Specifically, strict reserves should be established for the large WAs located in high elevations and surrounded by fewer people. Meanwhile for many small, highly fragmented and disturbed WAs, some sustainable management approaches with nature conservation as one of the primary management objectives might be optimal solutions.

## Methods

### Study area

**Figure 1:**
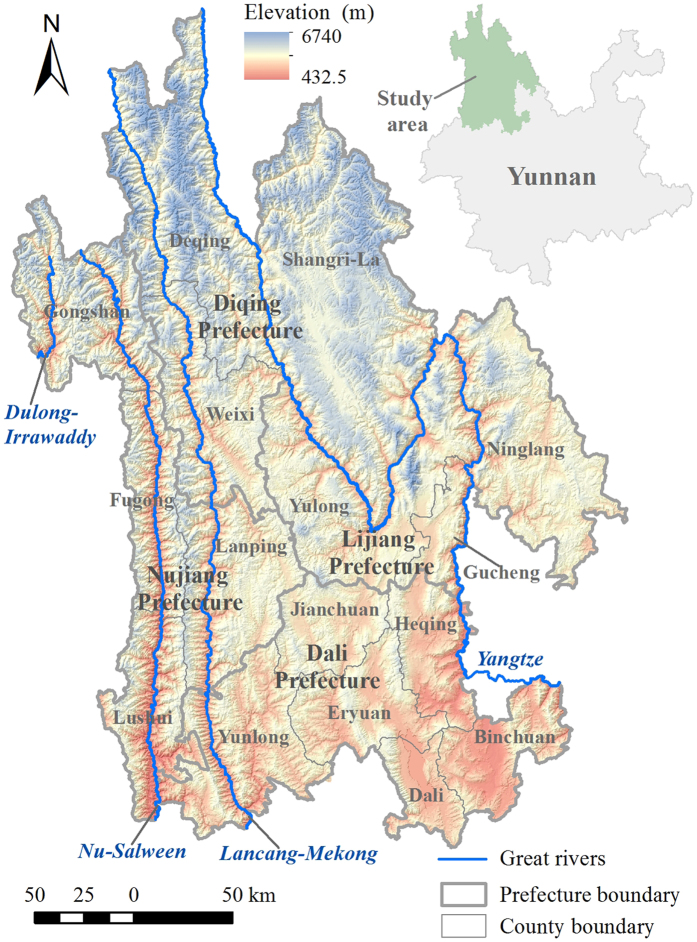
Three Parallel Rivers Region (TPRR) in southwest China. Different elevation spectrums overlaying on shaded relief were used to illustrate the region’s topography. The map was created using ESRI ArcGIS 10.0 (http://www.esri.com/).

**Figure 2:**
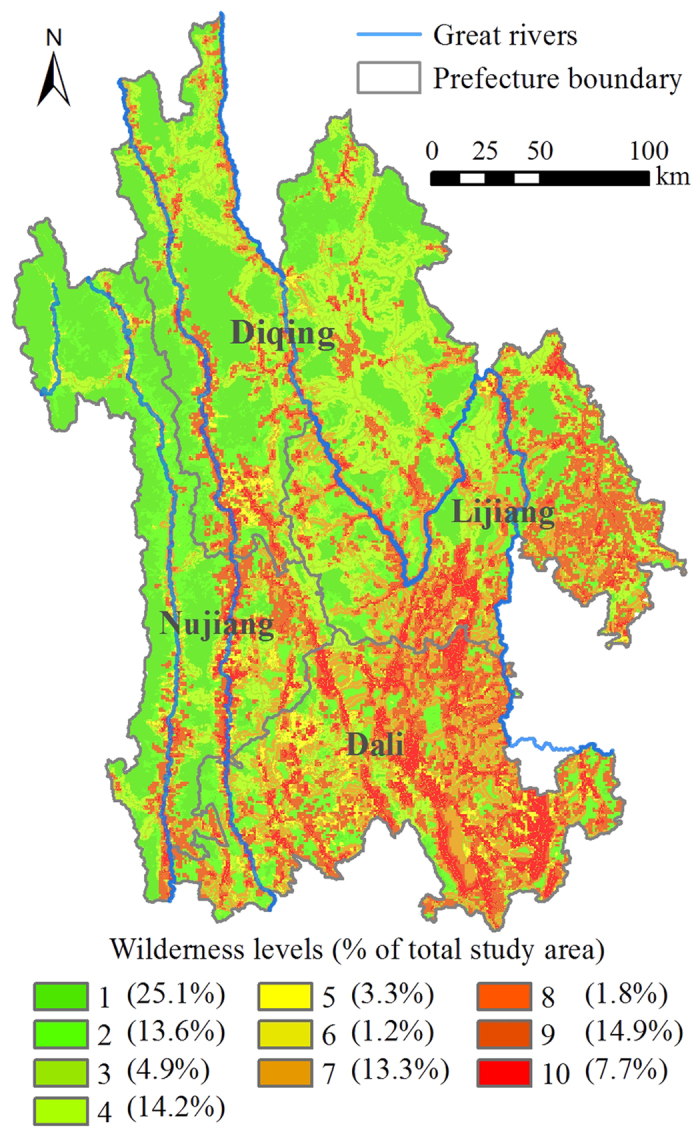
The spatial distribution of wilderness levels. The map was created using ESRI ArcGIS 10.0 (http://www.esri.com/).

**Figure 3:**
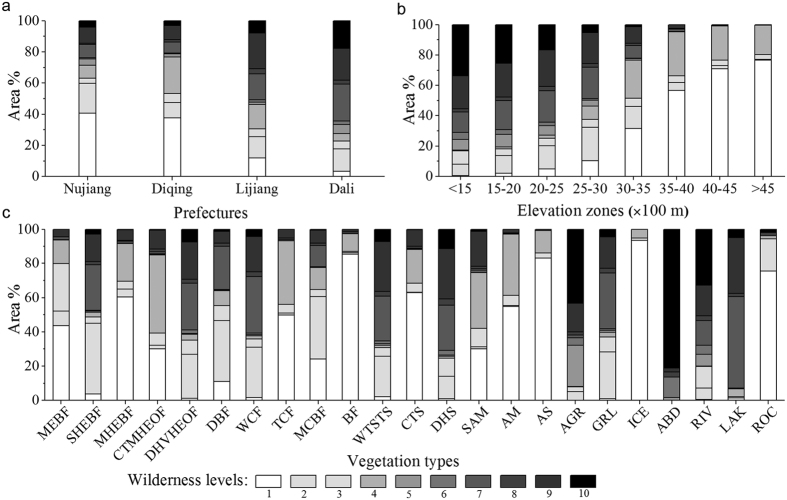
The area % of wilderness levels within different (**a**) prefectures, (**b**) elevation zones, and (**c**) vegetation types. Vegetation types: MEBF (monsoon evergreen broad-leaf forest), SHEBF (semi-humid evergreen broad-leaf forest), MHEBF (mid-montane humid evergreen broad-leaf forest), CTMHEOF (cold temperate montane hard-leaf evergreen oak forest), DHVHEOF (dry/hot valley hard-leaf evergreen oak forest), DBF (deciduous broad-leaf forest), WCF (warm conifer forest), TCF (temperate conifer forest), MCBF (mixed conifer broad-leaf forest), BF (bamboo forest), WTSTS (warm temperate sparse tree shrub), CTS (cold temperate shrub), DHS (dry/hot shrub), SAM (sub-alpine meadow), AM (alpine meadow), AS (alpine scree), AGR (agriculture), GRL (grassland), ICE (ice/snow), ABD (artificial building), RIV (rivers), LAK (lakes), and ROC (rock).

**Figure 4:**
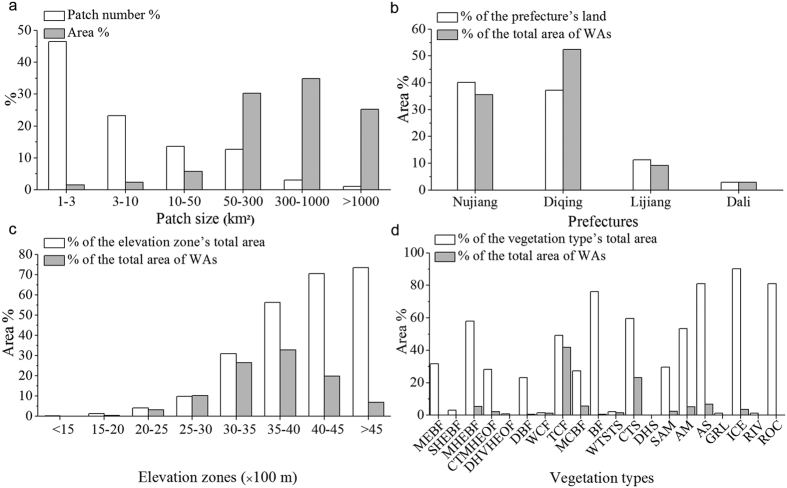
The spatial patterns of wilderness areas (WAs). (**a**) The area % (and patch number %) of different WAs patch size classes accounted for the total WAs area (and total patch number), and the distributions of WAs on different (**b**) prefectures, (**c**) elevation zones and (d) vegetation types (see Fig. 3 for the full names of vegetation types).

**Figure 5:**
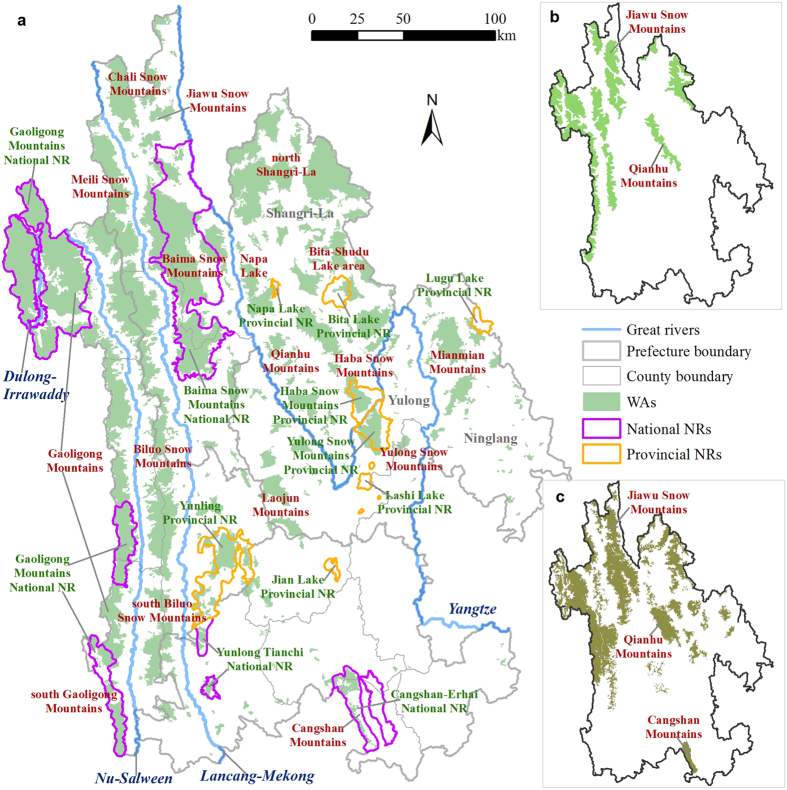
Comparisons between (a) wilderness areas (WAs) and (b) intact forest landscapes^20^ and (c) plant diversity priority conservation areas^24^, and the spatial distribution of nature reserves (NRs). The map was created using ESRI ArcGIS 10.0 (http://www.esri.com/).

**Figure 6:**
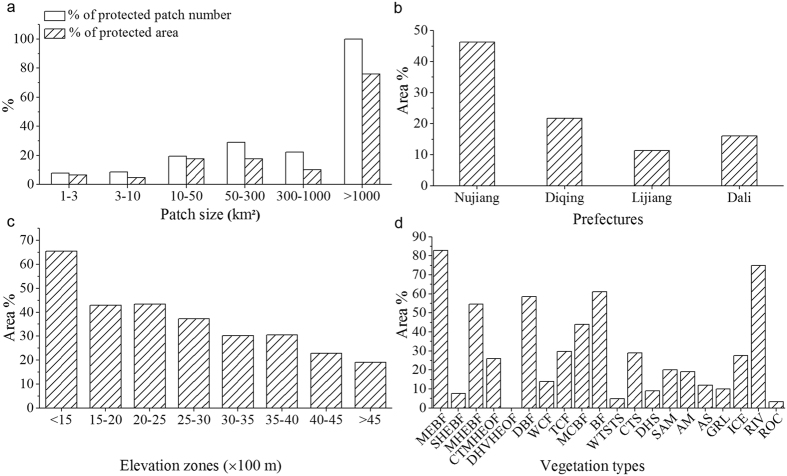
The conservation patterns of wilderness areas (WAs) by nature reserves (NRs). (**a**) % of the protected area (and protected patch number) accounted for the total area (and total patch number) within different WAs patch size classes, and the protection % of WAs that were located within different (**b**) prefectures, (**c**) elevation zones and (**d**) vegetation types (see Fig. 3 for the full names of vegetation types).

TPRR is situated in the southern Hengduan Montanans in northwest Yunnan of China (25°30′~29°15′ N, 98°05′ ~101°15′ E) and has a total area of 67,000 km^2^. TPRR also lies in Asia’s upstream area with three international rivers (i.e., Lancang-Mekong, Nu-Salween and Dulong-Irrawaddy) and Yangtze running in parallel from north to south (Fig. 1). TPRR is characterized by extremely high mountain ranges and deep parallel gorges, with higher elevations and more complex terrains in the northwest than in the southeast. TCF and alpine ecosystems are the major vegetation types in the north, while the south is dominated by the subtropical evergreen broad-leaf forest with SHEBF and WCF as the major types^27^. TPRR contains a complete altitudinal vegetation spectrum including subtropical, temperate, cold temperate, cold mountain, dry-hot valley, wetland and aquatic vegetation types^32^. Such unique natural conditions make TPRR a global biodiversity and cultural hotspot^27^. TPRR is one of the three largest centers for the origin and diversification of endemic species in China, and it harbors 30% and 24.3% of China’s total recorded higher plant and vertebrate species, respectively^39^. Eight major ethnic minorities inhabit in this region, including Tibetan, Naxi, Bai, Yi, Lisu, Pumi, Nu and Dulong. TPRR is administratively made up of 16 counties that belong to four prefectures of Nujiang, Diqing, Dali, and Lijiang (Fig. 1). Economic development significantly differs among regions and Dali is considered to be the most developed followed by Lijiang, Diqing and Nujiang^27^,^28^.

TPRR is an ideal study area for WAs identification since (1) it is recognized widely as a global biodiversity hotspot^39^ and WAs play an irreplaceable role in maintaining biodiversity at local scales^5^; (2) the WAs from global studies^20^,^21^,^22^ are not applicable at local settings and a systematic identification of WAs is still lacking to support on-ground conservation actions in TPRR; (3) the protection of WAs is under great pressure due to the unprecedented disturbances to local natural ecosystems by urbanization, road expansion, tourism development and other economic activities, which are driven by the western development strategy in China^27^,^39^.

### Mapping wilderness levels

WAs represent the baseline of natural ecosystems that are minimally influenced by human activities^6^. Thus, identifying WAs by using the indicators that can reflect the intensity of human disturbances is a common practice^8^. Referencing previous studies^10^,^16^,^22^, we selected five proxy indicators, including human population density, naturalness, fragmentation, remoteness and ruggedness, which were used to characterize the ecological features of WAs and the intensity of human disturbances on natural habitats.

Human population density has been widely used as an important indicator to measure the impacts of human activities on natural ecosystems^2^,^18^,^22^,^43^,^44^. Population density is assumed to be proportional to the resource demands in a region, which in turn determines the degree of disturbances and threats suffered by ecosystems^9^,^22^. We derived 11 levels of human disturbance scores by applying the scoring algorithm proposed by Sanderson *et al*.^22^. The disturbance scores for densities between 0 and 9 people per km^2^ decreased linearly from 11 to 2 and the score for densities ≥10 people per km^2^ was held constant at 1. The higher the score means the lower disturbance intensity being experienced by an ecosystem. We used the 2010 human population density grid data with a spatial resolution of 1 km × 1 km provided by the Data Center for Resources and Environmental Sciences, Chinese Academy of Sciences (RESDC; http://www.resdc.cn).

Naturalness reflects the degree to which an ecosystem has deviated from its original state due to human influence, and is a relative measure that depends on people’s understanding of the nature in specific spatial-temporal contexts^8^. Therefore, naturalness is often determined by assigning values to land-cover types^25^. Following the scoring criteria proposed by Carver *et al*.^16^ and also guided by expert knowledge, we assigned the naturalness scores ranging from 1 to 5 for various vegetation types, with higher scores indicating higher naturalness (Table 1). The experts we invited all have conducted studies regarding plants, wildlife, vegetations and biodiversity conservation in TPRR for many decades. The vegetation type map (scale 1: 100,000) was developed by the Institute of Ecology and Geobotany of Yunnan University^32^. We converted the naturalness layer into grid data with a spatial resolution of 100 m × 100 m, and then calculated the mean value for each central cell with a moving window of 3 × 3 cells, so that the naturalness of adjacent patches of different vegetation types could transit smoothly.

Ecological integrity was determined and gauged using the degree of habitat fragmentation as a critical proxy indicator^8^. Expansions of urban areas, agricultural lands, roads, and other human infrastructures are considered the direct factors leading to habitat fragmentation^51^. To measure fragmentation, we first merged the patches of natural vegetation types (except the types of AGR and ABD) into a single area, and then segmented the merged natural area with a road layer (including national, provincial, county, township and rural roads) by spatial overlaying analysis. After converting the layer into grid data with a resolution of 100 m × 100 m, we calculated for the effective mesh size by using the moving window function (window size 3 km × 3 km) in Fragstats 4.0^52^. Effective mesh size is a valid method for describing the habitat fragmentation in a region^51^. The road data was derived from the Yunnan Administrative Map (scale 1: 200,000) developed by the Mapping Institute of Yunnan Province^53^.

WAs are generally located in remote regions with very poor accessibility^10^,^11^. This study obtained the remoteness values by applying a spatial model proposed by Carver *et al*.^16^. Using the datasets on roads, digital elevation model (DEM) and land-use types as input information, we calculated the remoteness by running the path analysis tool with the Naismith’s Rule (see details in Carver *et al*.^16^) in ArcGIS 10.0 (ESRI Inc., 2010). The remoteness was calculated as the time period needed by humans to walk from any location to its adjacent road. The advantage of this model is that the effects of traffic conditions, distance, terrain and land cover types on human walking time are simultaneously considered. We used the 30 m Aster GDEM data^54^, and the land-use data (scale 1: 100,000) was provided by RESDC.

Human impacts are usually less in places with rugged terrains, and thus the remaining WAs are primarily distributed in mountainous regions^11^. This study used the terrain ruggedness index, generated by calculating the average difference in elevation between a center cell and its eight neighbor cells^55^.

The five indicator layers need to be normalized first due to their different measurement units and data ranges^56^. Studies have pointed out that once the effects of human activities on the habitats reach a certain threshold (e.g., limited distance effects of human residences and roads on ecosystems^29^), human disturbance will not be evident beyond that threshold^16^,^57^. Therefore, a logarithmic function^58^ was used to normalize the five layers and their normalized values ranged from 0 to 1.0.


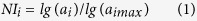


where *NI*_*i*_ is the normalized value, *a*_*i*_ is the cell value of indicator layer *i*, and *a*_*imax*_ is the maximum cell value of indicator layer *i*.

Due to lack of a priori knowledge about the relative effect of each indicator on wilderness, we weighted each indicator layer equally. We then applied principal component analysis (PCA) on these five normalized layers, and the first three axes accounted for 96.380% of the total Eigenvalues. Finally, TPRR was classified into 10 wilderness levels by running a cluster analysis on the first three PCA axes using the ISO Cluster Unsupervised Classification tool in ArcGIS 10.0. Wilderness levels decreased from level 1 to level 10 with level 1 being the highest. As proposed by Leu *et al*.^56^, we explored various cluster numbers by visually evaluating the classifications against the wilderness gradient in many reference locations, and found that the 10-level classification scheme had the best performance. We further classified the 10 wilderness levels into three broad categories of high (level 1–3), intermediate (level 4–7) and low (level 8–10) in order to investigate the spatial patterns of wilderness in a more general and clearer way.

To investigate the spatial patterns of wilderness, we calculated for the area proportions of 10 wilderness levels within different prefectures, elevation zones and vegetation types using spatial overlaying analyses. Following Wu^28^, we classified elevation zones as following: <1,500 m, 1,500–2,000 m, 2,000–2,500 m, 2,500–3,000 m, 3,000–3,500 m, 3,500–4,000 m, 4,000–4,500 m and >4,500 m. To explore the relationship between wilderness levels and elevations, we generated 10,000 random points across TPRR and extracted the elevation value and wilderness level for each point, and we then examined the Spearman’s Rank Correlation between wilderness levels and elevation values.

### Identifying WAs

WAs with conservation potentials should maintain their high naturalness and be large enough to sustain the long-term persistence of biodiversity^8^. Ferraz *et al*.^45^ showed that many bird species in habitat fragments of ≈1.0 km^2^ have the tendency to become extinct within one or two decades, while fragments >1.0 km^2^ only lose some species over a much longer time scale from several decades to a century. Therefore, we identified WAs as the patches that were characterized by the highest wilderness level 1 and ≥1.0 km^2^ in size. We then calculated for the area proportions of WAs located within different prefectures, elevation zones, and vegetation types. Subsequently, we compared the spatial distribution of our WAs with two previous conservation priority setting studies, including the intact forest landscapes^20^ and plant diversity priority conservation areas^24^.

### Assessing the conservation status of WAs

Spatial data on the national and provincial NR boundaries were derived from the “2014 annual report of nature reserves in Yunnan Province”^40^. We then measured the NR coverage for WAs in terms of different patch size classes, prefectures, elevation zones and vegetation types. In particular, we analyzed the shortfall in NR coverage relative to the Convention on Biological Diversity’s (CBD’s) Aichi Target 11, which “seeks to protect at least 17% of terrestrial and inland water areas”^42^.

## Additional Information

**How to cite this article**: Lin, S. *et al*. Identifying local-scale wilderness for on-ground conservation actions within a global biodiversity hotspot. *Sci. Rep.* **6**, 25898; doi: 10.1038/srep25898 (2016).

## Figures and Tables

**Table 1:** Naturalness of vegetation types in Three Parallel Rivers Region (TPRR).

Vegetation types	Naturalness	Vegetation types	Naturalness
Temperate conifer forest (TCF)	4	Dry/hot shrub (DHS)	3
Monsoon evergreen broad-leaf forest (MEBF)	4	Alpine meadow (AM)	4
Mid-montane humid evergreen broad-leaf forest (MHEBF)	4	Sub-alpine meadow (SAM)	4
Semi-humid evergreen broad-leaf forest (SHEBF)	3	Alpine scree (AS)	5
Cold temperate montane hard-leaf evergreen oak forest (CTMHEOF)	4	Grassland (GRL)	3
Mixed conifer broad-leaf forest (MCBF)	3	Ice/snow (ICE)	5
Deciduous broad-leaf forest (DBF)	3	Rock (ROC)	5
Warm conifer forest (WCF)	3	Rivers (RIV)	3
Dry/hot valley hard-leaf evergreen oak forest (DHVHEOF)	3	Lakes(LAK; ≥4,000 m)	5
Bamboo forest (BF)	4	Lakes (LAK; <4,000 m)	3
Cold temperate shrub (CTS)	5	Agriculture (AGR)	2
Warm temperate sparse tree shrub (WTSTS)	3	Artificial building (ABD)	1

Notes: Grasslands are mainly distributed in areas with elevations below 3,000 m and are strongly interfered by human activities, and thus were assigned a naturalness value of 3. As for some large lakes and rivers such as Erhai Lake, Lugu Lake, Dulong-Irrawaddy and Nu-Salween, they were scored by adopting the national water quality standards and the better the water quality was, the higher they were scored. The water quality data were derived from the “2013 annual bulletin of environmental conditions in Yunnan Province”^36^. In terms of some unnamed plateau lakes, those located in areas with elevations above 4,000 m were assigned a value of 5, while lakes located below elevations of 4,000 m were assigned with value 3.
